# Social capital and sleep disorders in Tibet, China

**DOI:** 10.1186/s12889-021-10626-x

**Published:** 2021-03-25

**Authors:** Wangla Ciren, Wanqi Yu, Qucuo Nima, Xiong Xiao, Junmin Zhou, Deji Suolang, Yajie Li, Xing Zhao, Peng Jia, Shujuan Yang

**Affiliations:** 1 Lhasa Chengguan District Center for Disease Control and Prevention, Lhasa, 850000 China; 2 West China School of Public Health and West China Fourth Hospital, Sichuan University, Chengdu, China; 3 Center for Disease Control and Prevention of Tibet autonomous region, Lhasa, 850000 China; 4 Department of Land Surveying and Geo-Informatics, The Hong Kong Polytechnic University, Hong Kong, China; 5 International Institute of Spatial Lifecourse Epidemiology (ISLE), Hong Kong, China

**Keywords:** Social capital, Sleep disorders, Tibet, Middle aged and elderly people

## Abstract

**Objective:**

Sleep plays an important role in the health and well-being of middle aged and elderly people, and social capital may be one of the important factors for sleep disorders. This study aimed to understand the relationship between social capital and sleep disorders in a unique region of China –Tibet that generally has the disadvantaged economic status compared to other parts of China.

**Methods:**

The study was based on Tibetan data from The China Multi-Ethnic Cohort (CMEC) and was conducted from May 2018 to September 2019. A total of 3194 Tibetans aged > 50 were selected from the community population by multi-stage stratified cluster sampling. Social capital was measured using two validated health-related social capital scales, family/community and society.. Sleep disorders were measured as the presence of disorders of initiating and maintaining sleep, early morning awakening, or daytime dysfunction. Logistic regression models were applied to examine the association between social capital and sleep disorders.

**Results:**

39.9% (1271/3194) of the participants had sleep disorders. In addition, after controlling for all potential variables, family social capital was significantly negatively associated with sleep disorders (OR = 0.95, *P* < 0.05), while community and society social capital was not associated with sleep disorders. Then, when we did all the sex-stratified analyses, the significant association between social capital and sleep disorders was found only in women (OR = 0.94, *P* < 0.05), while no association was found in males; neither males nor females showed any association with community and society social capital.

**Conclusion:**

Our study would help to better understand the extent of health inequality in China, and guide future interventions, strategies and policies to promote sleep quality in low-income areas, taking into account both the role of Tibetan specific cultural traditions, lifestyles and religious beliefs in social capital and the gender differences in social capital.

**Supplementary Information:**

The online version contains supplementary material available at 10.1186/s12889-021-10626-x.

## Introduction

Sleep disorders refer to conditions that affect sleep quality, timing, or duration and impact one’s ability to properly function while awake, such as insomnia, insufficient sleep (≤6 h/day), and excessive sleep (≥9 h/day) [1]. The awareness of the importance of sleep disorders in one’s health and well-being has been increasing [2, 3]. Previous studies have found that sleep disorders can cause multiple health problems [4], including psychological problems [5], endocrine disorders [6], chronic diseases [7, 8], and higher mortality rates [9]. It is estimated that the people older than 55 years, with nearly half having sleep disorders [10–14], were more likely to suffer from sleep disorders than their younger counterparts [15].

According to the socioecological model of sleep health [16], sleep disorders are driven by multiple contextual factors [1]. Much attention has been given to social determinants as they are relatively easier to be intervened. Social capital, embedded in the social fabric as an important social factor, is the network of social relations basing on social norms of trust and reciprocity that mobilize actors and coordinates and maintains their “field of presence” [17], which reflects the nature and quality of social connections among individuals in the community or social network [1]. According to the different scope and environment involved in the relationship network, it can be divided into family social capital and community and society social capital, which represent the resources obtained by individuals in the family or community and society, respectively [18]. Social capital may be one of the important factors for sleep disorders as the special role played by social capital in emotional perception and psychological state [19]. Poor social capital means that individuals cannot trust others to develop communication and thus cannot obtain enough resources to improve their life [20]. Perceived pressure and negative emotions further lead to depression and anxiety symptoms, which are closely related to sleep disorders [21]. However, most of the previous studies have been conducted in developed regions [22–25], and the association between social capital and sleep disorders may vary by context [26].

Few studies on the determinants of sleep disorders have been conducted in less developed regions, particularly in China. However, the burden of diseases in those regions is usually heavier than the one in better-off regions, so sleep disorders could double the health burden in the vulnerable regions. To fill this critical gap, this study aimed to study the association between social capital and sleep disorders in a unique region of China – Tibet, which generally has the disadvantaged economic status compared to other parts of China. Therefore, the findings of this study would help to better understand the extent of health inequalities in China and guide future interventions to promote the quality of sleep in low-income areas.

## Methods

### Study area

Our study was conducted in Tibet, China. Tibet, one of the five autonomous regions in China, sits in the southwest of the Qinghai-Tibet Plateau with the unique topography, natural climate, and traditional culture. However, due to the less developed economy compared to other western provinces, not to mention the eastern and central provinces of China, the per capita Gross Domestic Product (GDP) and average life expectancy in Tibet have been at the lowest level within the country [27, 28]. In 2017, the average life expectancy was only 70.6 years [29], about 10 years lower than that of developed regions in China (such as Beijing and Shanghai) [28]. Furthermore, Lamaism is popular and has become an important source of social capital in Tibet. The culture and social networks are closely related to religion, which makes the composition of social capital different from that of other regions in China [30, 31].

### Subject recruitment and data sources

The study was based on Tibetan data from The China Multi-Ethnic Cohort (CMEC) and was conducted from May 2018 to September 2019 [32]. A total of 3194 Tibetans over 50 years of age were recruited in our analysis. Participants were selected from the community population by multi-stage stratified cluster sampling. In the first stage, Lhasa and Aba Tibetan as minority settlements were selected as our research sites. In the second stage, the local centers for Disease Control and Prevention (CDCs) selects several communities (depending on the size of the communities) in the settlement, taking into account the immigration status, local health conditions, and ethnic structure. In the final stage, all subjects who met the inclusion criteria were invited to participate in our study. The inclusion criteria we used included (I) over 50 years of age on the day of survey, since the average life expectancy was lower in Tibetan and limited samples were recruited above 60 years; (II) permanent residents, the capability of completing baseline surveys and the availability to complete follow-up studies; and (III) complete questionnaire interviews. The exclusion criteria were as follows: (I) inability to provide a unique national identity card; (II) severe physical or mental illness (such as schizophrenia and bipolar disorder); and (III) refusal to comply with study requirements. All participants signed informed consent before data collection. Ethical approval was received from the Sichuan University Medical Ethical Review Board (K2016038).

The survey consists of an electronic questionnaire and face-to-face interviews, and the questionnaire items used in this study are provided in Supplementary File 1. Collaborative and field workflow CMEC baseline survey was conducted through the collaboration of many institutions, including academic institutions, CDCs, local clinical centers, Third-Party Medical Laboratories (TPML) companies and local governments. Each agency has a clear role to play in the investigation process. Usually, the local government started the CMEC propaganda or publicity activities a few weeks before the formal investigation. At the same time, residents fully informed the benefits and requirements of participating in the CMEC study. We used a tablet with a self-developed application (CMEC APP) to collect questionnaire information. The information was collected in face-to-face interviews by trained interviewers who were local college students with a medical background. For a skilled interviewer, it usually takes 30 to 45 min to complete a questionnaire survey.

Strict quality control was carried out and the whole process can be divided into two parts: detection and verification. In the detection part, we check the duplication, completeness, outliers and logic errors by predesigned algorithms. Once suspicious data errors were detected, we will conduct further verification process by listening to the audio recording, reviewing the inspection report or calling the participants back. Then, the data were revised and the whole correction process was recorded by our computer system.

### Measures of key variables

#### Measurement of social capital

Social capital was measured using a validated Chinese version of Health-related Social Capital Measurement [33, 34]. According to the living and environmental characteristics of the middle-aged and old people in Tibet, and more other variables collection and survey duration, family social capital, community and society social capital scales were considered in this questionnaire.

Family social capital scale was assessed using the following two items: 1) “you receive empirical support from family”, 2) “you always receive financial support from family”. Community and society social capital scale using three items, including: 1) “ you frequently participated in activities organized by community organizations in the last year “, 2) “ You always received support from community organizations in the last year “, 3) “ You have been treated fairly by society”. The answer categories ranged from a scale of 1 (strongly disagree) to 5 (strongly agree), with higher total scores, indicating stronger social capital (Supplementary File 2). Cronbach’s alpha, ranging from 0 to 1, was used to assess the reliability of social capital scales. A Cronbach’s alpha of 0.6 or greater was considered acceptable [35, 36]. The Cronbach’s α value of the social capital scale was 0.640.

Measurement of sleep disorders.

Three types of sleep disorders, i.e., disorders of initiating and maintaining sleep (DIMS), early morning awakening (EMA), and daytime dysfunction (DDF), were measured using a questionnaire from China Kadoorie Biobank By asking participants to answer “yes” or “no” to the three questions [37], respectively: 1) Did you have difficulty in falling asleep (sleep onset latency ≥30 min), or wake up in the middle of the night at least three days a week?; 2) Did you wake up too early in the morning and have difficulty falling asleep again at least three days a week; 3) Did you have trouble keeping sober-minded during daytime due to poor sleep at least three days a week? Participants were classified as sleep disorders if they answered “yes” to any of the three questions.

Demographic and health characteristics.

Participants were asked about their demographic and health characteristics, including age, Hukou (residential status), sex, marital status, commercial insurance status, education level, annual household income (in RMB), occupation, smoking and drinking status, Body Mass Index (BMI), history of hypertension and diabetes, and mental status (depression and anxiety). The characteristics of all participants were considered as potential covariates.

### Statistical analyses

Statistical analysis, descriptive analysis and multiple logistic regression analysis were used in this study. First, each variable can be divided into “Yes - sleep disorders” and “No - sleep disorders”, categorical variables were expressed as frequencies and percentages, and continuous variables were expressed as mean ± standard deviation. Different hypothesis test methods (e.g., t-test, Chi-square test, Fisher’s exact test) were used to test the differences between “Yes - sleep disorders” and “No - sleep disorders” for variables of different distribution types and report the *p* value. The univariate logistic regression model (Model 1) was used to examine the association between the independent variables (including social capital) and the dependent variable (sleep disorder). Second, all demographic and health characteristics that were marginally significant in univariate analysis were taken into account as covariates. Then social capital, sleep disorders and all covariates were included in the multivariate logistic regression models step by step (Model 2 to 5). Model 5 takes into account all potential covariates, including demographic features (i.e., age, Hukou, sex, marital status), Socioeconomic Status (i.e., educational level, annual household income, occupation and commercial insurance status), health behaviors (i.e., smoking and drinking), and health-related variables (i.e., BMI, history of hypertension, history of diabetes, depression and anxiety).

The association was calculated by odds ratio (OR) and 95% confidence interval (CI). All data were analyzed by R 3.6.2. Statistical significance was declared if a two-sided *p*-value was less than 0.05.

## Results

### Descriptive statistics of the participants

A total of 3194 Tibetans over 50 years of age were included in our analysis. The mean age of the study participants was 59.1 ± 7.2 years, and most of them were rural (84.8%), female (59.0%), married/cohabitation (86.6%), had no formal education (62.5%), unemployed (63.6%), had an annual family income of 12,000–59,999 yuan and no commercial insurance (96.4%). Of these, 76.7% had never smoking and 88.2% had never drinking. Only 2.4% of middle-aged and elderly Tibetans had symptoms of depression, and 2.6% of them had symptoms of anxiety. The other demographic and disease-related data of the study participants are shown in Table 1.

**Table 1:** Baseline characteristics of the participants and their crude associations with sleep disorders

Variables	Mean ± SD or Number (percentage, %)			*p*-value	Odds ratio
	Overall (*n* = 3194)	With sleep disorders (*n* = 1271)	Without sleep disorders (*n* = 1923)		
Age (year)	59.1 ± 7.2	59.7 ± 7.2	58.6 ± 7.2	***p*** **< 0.001**	1.02 (1.01, 1.03)^***^
Sex				***p*** **< 0.001**	
Male	1308 (41.0)	411 (32.3)	897 (46.6)		1.00 (ref.)
Female	1886 (59.0)	860 (67.7)	1026 (53.4)		1.83 (1.58, 2.12)^***^
Hukou				***p*** **< 0.001**	
Urban	486 (15.2)	262 (20.6)	224 (11.6)		1.00 (ref.)
Rural	2708 (84.8)	1009 (79.4)	1699 (88.4)		0.51 (0.42, 0.62)^***^
Marital status				***p*** **< 0.001**	
Unmarried	83 (2.6)	21 (1.7)	62 (3.2)		1.00 (ref.)
Separated/divorced/widowed	344 (10.8)	165 (13.0)	179 (9.3)		2.72 (1.59,4.66)^***^
Married/cohabitation	2767 (86.6)	1085 (85.4)	1682 (87.5)		1.90 (1.15, 3.14)^*^
Educational level				***p*** **< 0.001**	
No formal	1997 (62.5)	850 (66.9)	1147 (59.6)		1.00 (ref.)
Primary school	972 (30.4)	330 (26.0)	642 (33.4)		0.69 (0.59, 0.81)^***^
Junior high school or above	225 (7.0)	91 (7.2)	134 (7.0)		0.92 (0.69, 1.21)
Annual household income (Chinese *yuan*)				***p*** **< 0.05**	
< 12,000	824 (25.8)	339 (26.7)	485 (25.2)	***p*** **< 0.05**	1.00 (ref.)
12,000-59,999	1864 (58.4)	701 (55.2)	1163 (60.5)		0.86 (0.73, 1.02)
60,000-99,999	258 (8.1)	116 (9.1)	142 (7.4)		1.17 (0.88, 1.55)
> 100,000	245 (7.7)	114 (9.0)	131 (6.8)		1.25 (0.93, 1.66)
Occupation				***p*** **< 0.001**	
Peasant	339 (10.6)	131 (10.3)	208 (10.8)		1.00 (ref.)
Employed	543 (17.0)	192 (15.1)	351 (18.3)		0.87 (0.66, 1.15)
Unemployed	2031 (63.6)	795 (62.6)	1236 (64.3)		1.02 (0.81, 1.29)
Retired	279 (8.7)	151 (11.9)	128 (6.7)		1.87 (1.36, 2.58)^**^
Commercial insurance status				***p*** **< 0.001**	
No	3076 (96.4)	1205 (95.0)	1871 (97.3)		1.00 (ref.)
Yes	116 (3.6)	64 (5.0)	52 (2.7)		1.91 (1.32, 2.78)^*^
Smoking status				***p*** **< 0.001**	
Never	2450 (76.7)	1047 (82.4)	1403 (73.0)		1.00 (ref.)
Former	221 (6.9)	75 (5.9)	146 (7.6)		0.69 (0.52, 0.92)^*^
Current	522 (16.3)	149 (11.7)	373 (19.4)		0.54 (0.44, 0.66)^**^
Drinking status				***p*** **< 0.001**	
Never	2816 (88.2)	1155 (90.9)	1661 (86.4)		1.00 (ref.)
Former	91 (2.8)	42 (3.3)	49 (2.5)		1.23 (0.81, 1.87)
Current	286 (9.0)	74 (5.8)	212 (11.0)		0.50 (0.38, 0.66)^**^
Body mass index				***p*** **< 0.001**	
< 24	626 (22.9)	272 (23.9)	354 (22.1)		1.00 (ref.)
[24,28)	1569 (57.3)	609 (53.6)	960 (60.0)		0.83 (0.68, 1.00)^*^
≥ 28	542 (19.8)	255 (22.4)	287 (17.9)		1.16 (0.92, 1.46)
History of hypertension				0.24	
No	2192 (69.7)	857 (67.4)	1335 (69.5)		1.00 (ref.)
Yes	1001 (31.3)	414 (32.6)	587 (30.5)		1.10 (0.94, 1.28)
History of diabetes				0.49	
No	3080 (96.5)	1222 (96.1)	1858 (96.7)		1.00 (ref.)
Yes	113 (3.5)	49 (3.9)	64 (3.3)		1.16 (0.80, 1.70)
Depression				***p*** **< 0.001**	
No	3110 (97.6)	1219 (95.9)	1891 (98.6)	***p*** **< 0.001**	1.00 (ref.)
Yes	78 (2.4)	52 (4.1)	26 (1.4)		3.10 (1.93, 5.00)^**^
Anxiety				***p*** **< 0.001**	
No	3104 (97.4)	1214 (95.5)	1890 (98.6)		1.00 (ref.)
Yes	84 (2.6)	57 (4.5)	27 (1.4)		3.29 (2.07, 5.22)^**^
Family social capital	8.1 ± 2.1	8.1 ± 2.2	8.2 ± 2.0	0.42	0.97 (0.93, 1.00)^*^
Community/Society social capital	10.1 ± 2.0	10.0 ± 2.0	10.1 ± 2.0	0.64	0.98 (0.94, 1.01)

Bolden numbers indicate statistical significance (^*^p < 0.05, ^**^p < 0.01, ^***^p < 0.001)

The median scores for family SC and community and society SC were 8 and 10, respectively. The scores of each item of social capital can be seen in Supplementary File 2 Of all participants, 39.9% of them had sleep disorders, 69.9% had DIMS, 73.0% had EMA, and 88% had DDF (Supplementary File 3).

### Association between social capital and sleep disorder

The distribution of all variables in “Yes - sleep disorders” and “no - sleep disorders” were statistically significant. Univariate logistic regression analysis showed that age, Hukou, sex, marital status, educational level, occupation, commercial insurance status, smoking and drinking status, and mental health were significantly associated with sleep disorders (*p* < 0.05) (Table 1).

There was no significant association between family social capital and sleep disorders when only taking family social capital as an independent variable; after adjusting for demographic features, socioeconomic status, health behavior and health-related variables (BMI, history of hypertension, diabetes, depression and anxiety), family social capital and sleep disorders showed significant negative association, and the higher the score of social capital, the fewer sleep disorders (OR = 0.95, *P* < 0.05). However, there is no significant association between community and social capital, whether it was adjusted or not (Table 2).

**Table 2:** Associations between social capital and sleep disorders

	OR (95%CI)				
	Model 1	Model 2	Model 3	Model 4	Model 5
Family social capital	**0.97** (0.93, 1.00)^*^	**0.96** (0.93, 1.00)^*^	**0.96** (0.93, 1.00)^*^	**0.96** (0.93, 1.00)^*^	**0.95** (0.91, 0.98)^**^
Community/Society social capital	0.98 (0.94, 1.01)	0.98 (0.95, 1.02)	0.98 (0.94, 1.02)	0.98 (0.94, 1.02)	0.98 (0.94, 1.02)

Bolden numbers indicate statistical significance (^*^p < 0.05, ^**^p < 0.01, ^***^p < 0.001)

Model 1: crude model (without adjustment); Model 2: Model 1 adjusted for demographic features (i.e., age, sex, Hukou, marital status);

Model 3: Model 2 adjusted for socioeconomic status (i.e., educational level, annual household income, occupation, commercial insurance status);

Model 4: Model 3 adjusted for behavioral factors (i.e., smoking status, drinking status);

Model 5: Model 4 adjusted for health conditions (i.e., body mass index, history of hypertension, history of diabetes, depression, anxiety)

When performing stratified analysis by sex, family social capital was negatively associated with sleep disorders in females (OR = 0.94, P < 0.05), while no association was found in males; neither males nor females showed any association with community and society social capital (Table 3).

**Table 3:** Associations between all influential factors and sleep disorders in two groups of models with the family social capital (FSC) and community/society social capital (CSC) as the main predictor variable separately

Characteristic	FSC as the main predictor variable			CSC as the main predictor variable		
	Overall	Male	Female	Overall	Male	Female
FSC	0.95 (0.91, 0.98)^*^	0.95 (0.90, 1.01)	0.94 (0.90, 0.99)^*^	–	–	–
CSC	–	–	–	0.98 (0.94, 1.02)	1.01 (0.94, 1.08)	0.96 (0.91, 1.01)
Age (year)	1.02 (1.00, 1.03)^*^	1.02 (1.00, 1.04)	1.02 (1.00, 1.03)^*^	1.02 (1.00, 1.03)^*^	1.02 (1.00, 1.04)	1.02 (1.00, 1.03)
Sex						
Male	1.00 (ref.)	–	–	1.00 (ref.)	–	–
Female	1.6 (1.32, 1.95)^***^	–	–	1.60 (1.32, 1.95)^***^	–	–
Hukou						
Urban	1.00 (ref.)	1.00 (ref.)	1.00 (ref.)	1.00 (ref.)	1.00 (ref.)	1.00 (ref.)
Rural	0.71 (0.55, 0.93)^*^	0.59 (0.37, 0.93)^*^	0.78 (0.56, 1.08)	0.72 (0.55, 0.93)^*^	0.59 (0.37, 0.93)^*^	0.80 (0.57, 1.11)
Marital status						
Unmarried	1.00 (ref.)	1.00 (ref.)	1.00 (ref.)	1.00 (ref.)	1.00 (ref.)	1.00 (ref.)
Separated/divorced/widowed	3.29 (1.76, 6.17)^***^	2.60 (0.80, 8.4)	3.38 (1.57, 7.26)	3.18 (1.70, 5.96)^***^	2.52 (0.78, 8.10)	3.29 (1.53, 7.08)^***^
Married/cohabitation	2.84 (1.58, 5.09)^***^	2.70 (0.99, 7.34)	2.79 (1.35, 5.76)^*^	2.73 (1.53, 4.89)^***^	2.59 (0.96, 7.00)	2.70 (1.31, 5.57)^*^
Educational level						
No formal	1.00 (ref.)	1.00 (ref.)	1.00 (ref.)	1.00 (ref.)	1.00 (ref.)	1.00 (ref.)
Primary school	0.83 (0.69, 1.00)	0.87 (0.64, 1.17)	0.82 (0.64, 1.03)	0.83 (0.69, 1.00)	0.86 (0.64, 1.16)	0.81 (0.64, 1.03)
Junior high school or above	0.77 (0.55, 1.08)	0.85 (0.53, 1.38)	0.73 (0.45, 1.21)	0.77 (0.55, 1.08)	0.86 (0.53, 1.38)	0.73 (0.45, 1.21)
Annual household income (Chinese *yuan*)						
< 12,000	1.00 (ref.)	1.00 (ref.)	1.00 (ref.)	1.00 (ref.)	1.00 (ref.)	1.00 (ref.)
12,000-59,999	0.84 (0.7, 1.02)	0.79 (0.57, 1.11)	0.88 (0.69, 1.11)	0.84 (0.70, 1.02)	0.78 (0.56, 1.09)	0.88 (0.69, 1.11)
60,000-99,999	0.92 (0.67, 1.26)	0.73 (0.43, 1.24)	1.06 (0.70, 1.59)	0.92 (0.67, 1.26)	0.73 (0.43, 1.25)	1.05 (0.70, 1.58)
> 100,000	0.95 (0.67, 1.35)	0.75 (0.43, 1.30)	1.14 (0.72, 1.80)	0.96 (0.68, 1.36)	0.75 (0.43, 1.31)	1.15 (0.73, 1.82)
Occupation						
Peasant	1.00 (ref.)	1.00 (ref.)	1.00 (ref.)	1.00 (ref.)	1.00 (ref.)	1.00 (ref.)
Employed	1.00 (0.72, 1.37)	0.85 (0.54, 1.32)	1.15 (0.71, 1.85)	1.00 (0.73, 1.38)	0.86 (0.55, 1.35)	1.13 (0.70, 1.82)
Unemployed	0.88 (0.68, 1.15)	0.71 (0.47, 1.07)	0.99 (0.70, 1.4)	0.88 (0.68, 1.15)	0.71 (0.47, 1.08)	0.99 (0.70, 1.40)
Retired	1.36 (0.89, 2.09)	1.35 (0.71, 2.54)	1.17 (0.65, 2.11)	1.39 (0.91, 2.12)	1.37 (0.73, 2.58)	1.19 (0.66, 2.14)
Commercial insurance status						
No	1.00 (ref.)	1.00 (ref.)	1.00 (ref.)	1.00 (ref.)	1.00 (ref.)	1.00 (ref.)
Yes	1.77 (1.17, 2.69)^*^	1.41 (0.71, 2.80)	2.09 (1.22, 3.57)^*^	1.75 (1.15, 2.65)^*^	1.37 (0.69, 2.72)	2.07 (1.21, 3.54)^*^
Smoking status						
Never	1.00 (ref.)	1.00 (ref.)	1.00 (ref.)	1.00 (ref.)	1.00 (ref.)	1.00 (ref.)
Former	1.00 (0.71, 1.40)	1.03 (0.71, 1.50)	0.97 (0.40, 2.35)	1.02 (0.72, 1.43)	1.05 (0.72, 1.53)	0.99 (0.41, 2.40)
Current	0.76 (0.59, 0.98)^*^	0.83 (0.60, 1.14)	0.61 (0.38, 0.97)^*^	0.76 (0.59, 0.99)^*^	0.83 (0.60, 1.14)	0.60 (0.38, 0.96)^*^
Drinking status						
Never	1.00 (ref.)	1.00 (ref.)	1.00 (ref.)	1.00 (ref.)	1.00 (ref.)	1.00 (ref.)
Former	1.08 (0.68, 1.73)	1.41 (0.75, 2.68)	0.81 (0.41, 1.6)	1.10 (0.69, 1.75)	1.43 (0.76, 2.71)	0.80 (0.41, 1.58)
Current	0.73 (0.53, 0.99)^*^	0.77 (0.53, 1.11)	0.65 (0.37, 1.15)	0.73 (0.54, 1.00)	0.77 (0.53, 1.12)	0.66 (0.37, 1.16)
Body mass index						
< 24	1.00 (ref.)	1.00 (ref.)	1.00 (ref.)	1.00 (ref.)	1.00 (ref.)	1.00 (ref.)
[24,28)	0.84 (0.69, 1.02)	0.75 (0.55, 1.04)	0.90 (0.70, 1.16)	0.84 (0.69, 1.02)	0.76 (0.55, 1.05)	0.89 (0.69, 1.15)
≥ 28	1.02 (0.80, 1.30)	0.78 (0.51, 1.19)	1.20 (0.88, 1.63)	1.03 (0.80, 1.31)	0.79 (0.52, 1.21)	1.20 (0.88, 1.63)
History of hypertension						
No	1.00 (ref.)	1.00 (ref.)	1.00 (ref.)	1.00 (ref.)	1.00 (ref.)	1.00 (ref.)
Yes	1.09 (0.91, 1.29)	0.96 (0.72, 1.29)	1.17 (0.93, 1.46)	1.09 (0.92, 1.30)	0.96 (0.72, 1.29)	1.18 (0.94, 1.47)
History of diabetes						
No	1.00 (ref.)	1.00 (ref.)	1.00 (ref.)	1.00 (ref.)	1.00 (ref.)	1.00 (ref.)
Yes	1.05 (0.68, 1.61)	1.30 (0.71, 2.36)	0.90 (0.48, 1.66)	1.04 (0.68, 1.59)	1.27 (0.7, 2.32)	0.9 (0.48, 1.67)
Depression						
No	1.00 (ref.)	1.00 (ref.)	1.00 (ref.)	1.00 (ref.)	1.00 (ref.)	1.00 (ref.)
Yes	1.98 (1.09, 3.58)^*^	2.34 (0.85, 6.47)	1.83 (0.88, 3.84)	1.94 (1.07, 3.50)^*^	2.29 (0.83, 6.34)	1.79 (0.86, 3.73)
Anxiety						
No	1.00 (ref.)	1.00 (ref.)	1.00 (ref.)	1.00 (ref.)	1.00 (ref.)	1.00 (ref.)
Yes	2.16 (1.23, 3.80)^*^	1.51 (0.46, 4.89)	2.45 (1.27, 4.74)^*^	2.17 (1.24, 3.81)^*^	1.53 (0.47, 4.97)	2.44 (1.27, 4.72)^*^

Bolden numbers indicate statistical significance (^*^p < 0.05, ^**^p < 0.01, ^***^p < 0.001)

## Discussion

This study aimed to understand the level of social capital and the prevalence of sleep disorders in middle-aged and elderly people in Tibet, China, and the effect of social capital on sleep disorders. The results showed that 39.9% (1271 / 3194) of the participants had sleep disorders. Besides, family social capital was significantly negatively associated with sleep disorders, while community and social capital were not. In particular, even after controlling for all potential variables, family social capital was negatively associated with sleep disorders (OR = 0.95, *P* < 0.05). Then, when we did all the sex-stratified analyses, the significant association between social capital and sleep disorders was found only in women.

The prevalence of sleep disorders among middle-aged and elderly people in Tibet is higher than in other regions in China [38–40], but similar to the remote, high-altitude, low-income areas of Xinjiang and Qinghai [41–43]. On the one hand, the high prevalence of sleep disorders may be explained by a comparatively limited special geographical environment. Tibet is known as the “Third Pole”, with an average altitude of 4500 m, high altitude and low oxygen environment in previously shown to be associated with an increased risk of sleep disorders in China [44, 45]. On the other hand, the topography of Tibet is complex and rugged, which the challenges exercise performance of residents during work and leisure activities [46]. Moderate and reasonable exercise is considered to be helpful in sleep quality [47]. Finally, 84.8% of the Tibetans were in rural areas and 63.6% were unemployed. These socio-economic factors may be related to poor sleep quality in previous studies [48, 49].

Only family social capital and sleep disorders showed a significant negative association, and similar results also have been found in East Asia, including China [19], Japan [50] and South Korea [51]. A study conducted by Rebecca and colleagues in Philadelphia and neighboring counties in the United States found that poor sleep quality in adults (e.g., short or long sleep, moderate-to-severe insomnia) was associated with poor social capital (e.g., fewer group memberships, less neighborhood helping behavior, belonging, trust, and improvement) [1]. In Tibet, the traditional customs, family culture and religious characteristics are very unique, different from other places, which may the reasons for the association between family social capital and sleep disorders. Most Tibetans were nomads in the past [52]. Nomadism is a lifestyle in arid grassland areas that utilize water and grass resources by mobile grazing on horseback to obtain living materials [53]. Although some Tibetans have moved from “nomadism” to “settlement” in recent years [54], most of them still adhere to ancient and traditional lifestyles [55]. This unique way of living and production is based on the protection of scarce water resources and grasslands [52], which makes them more difficult to develop stable communities and social relationships outside the family, and makes the social capital within the family more important. Also, Tibet is a scarcely populated area with inadequate transportation networks [56], which makes family ties closer than community and social ties. In the study of social support for the elderly in Xigaze, Tibet, it is found that kinship plays a prominent role in the social support system, while non-relatives such as neighbors, friends and colleagues are on the periphery of their social support network and only play a marginal role [57]. Social support represents the size and source of social networks of people helping others and is closely related to social capital [58]. Finally, almost all the people in Tibet believe in Lamaism [30]. Religion is an important component of social capital, and its doctrines have a close influence on believers [31]. Lamaism values family moral education and regards family harmony and mutual assistance as a practice [59], which makes Tibetans pay more attention to the maintenance of family relations.

In our study, the sleep quality of middle-aged and elderly women in Tibet is more sensitive to lower family social capital. Perhaps due to the influence of Tibetan traditional culture, women’s family status is lower than that of men, and they usually need to take care of children and carry out unpaid housework [60]. Moreover, women tend to establish emotional and time-consuming kinship between family members and close friends, and also tend to spend more time communicating with family members, which creates fewer friendships or social networks with thick trust in women [61]. Our survey results show that the unemployment rate of middle-aged and elderly women in Tibet is 75.2%, which is far higher than that of 47% of men. Unemployment can lead to smaller social networks and lower social capital outside the family, which is also related to poor sleep quality [49]. Finally, this result may be partly explained by the female-specific physiological state. The average postmenopausal age of Tibetan women is 45 years old [62]. The specific postmenopausal state and/or hormonal effects before and after menopause may also lead to sleep disorders [63].

Our study is based on data from CMEC, which is the first large-scale cohort study focusing on ethnic minority groups in China, and standardizes survey methods and carried out multiple strict quality control measures. However, we also have some limitations. First, considering that there are many variables in the questionnaire survey, it is not enough to observe only three dimensions of social capital. The later cohort follow-up should consider more dimensions of social capital. Second, this study is a cross-sectional study, which may prevent the identification of a causal association between social capital and sleep disorders [64, 65]. Third, Tibetan samples are mainly concentrated in Lhasa residents and Aba herdsmen above 50 years of age, which may not represent the middle-aged and elderly people of the whole Tibet region. Finally, considering that there are great disparities in the natural environment of Tibetan, further studies still need to include more participants for further analysis [66, 67].

## Conclusions

Our findings demonstrated that the lower level of family social capital of middle-aged and elderly people, especially among females, is associated with sleep disorders. Social capital plays a role in the sleep disorders of middle-aged and elderly Tibetans. Improving their social capital may be able to alleviate sleep disorders, improve quality of life and prolong life expectancy. When designing interventions, strategies and policies to promote sleep-related health behaviors, we should not only consider the role of Tibetans’ special cultural traditions, lifestyle and religious beliefs in social capital, but also pay attention to sex differences in social capital. In Tibetan, population-based and prospective studies of the sex gap in social capital and sleep disorders are worth conducting, toward the promotion of positive health-related behaviors.

## Supplementary Information

**Additional file 1.**

**Additional file 2: Supplementary File 2.** Measurements of the social capital of the participants.

**Additional file 3: Supplementary File 3.** Conditions of sleep disorders of the participants.

## Data Availability

The quantitative data used to support the findings of this study were supplied by The China Multi-Ethnic Cohort (CMEC) research group under license and so cannot be made freely available. Requests for access to these data should be made to Shujuan Yang, rekiny@126.com.

## Abbreviations

BMI: Body Mass Index
OR: Odds ratio
CI: Confidence interval
CMEC: China Multi-Ethnic Cohort
