# Supplementary material for: Social capital and sleep disorders in Tibet, China
Source: BMC Public Health. 2021 Mar 25;21:591. doi: 10.1186/s12889-021-10626-x (PMC7992333; doi:10.1186/s12889-021-10626-x)
Supplement: Supplementary file 1 — Additional file 1. [file 12889_2021_10626_MOESM1_ESM.docx]

**Supplementary File 1.**

**The China Multi-Ethnic Cohort Study (CMEC)**

【Baseline Questionnaire】

**Name:**

**Mobile telephone:**

**Home address:**  City

District/County

Street/Village

**ID:** 🞎🞎🞎🞎🞎🞎🞎🞎🞎🞎🞎🞎🞎🞎

；

**Section 1: Background information**  **A1 Sex:** 🞎Male  🞎Female 

**A2 National ID number:** 🞎🞎🞎🞎🞎🞎🞎🞎🞎🞎🞎🞎🞎🞎🞎🞎🞎🞎

**A3 What is your current registered permanent residence?**

🞎Rural 🞎Urban 🞎 Unified household

**A4 What is your current marital status?**

🞎Married / cohabiting 🞎Widowed

🞎Separated */* divorced 🞎Never married

**A5 What is the highest level of school education you ever received?**

🞎No formal school 🞎Primary School

🞎Middle School 🞎High School / Technical School

🞎College 🞎University

**A6 What is your current occupation?**

🞎Agriculture & related workers 🞎 Factory worker

🞎Administrator / manager 🞎Professional / technical

🞎Sales & service workers 🞎 Retired

🞎House wife / husband 🞎Self-employed

🞎Unemployed 🞎Other or not stat

**A7 Do you have commercial insurance?**

🞎YES

🞎NO

**A8 What is the total income last year in your household?**

🞎＜12000yuan

🞎12000-19999yuan

🞎20000-59999yuan

🞎60000-99999yuan

🞎100000-199999yuan

🞎≥200000yuan

**A9 weight:**🞎🞎 **kg** (Field measurements)

**A10 height:**🞎🞎 **m** (Field measurements)

**Section 2: Smoking history**

**B1 Do you smoke?**

🞎never (smoked less than 100 cigarettes during their life)

🞎ever (not currently smoking)

🞎current (smoking at least one cigarette or less than one cigarette a day)

Section 3: Alcohol consumption

**C1 During the past 12 months, how often did you drink any alcohol?**

🞎Never or almost never

🞎Only occasionally

🞎Only at certain seasons

🞎Every month but less than weekly

🞎Usually at least once a week

Section 4: Personal & family medical history

**D1 Has a doctor EVER told you that you had had the following disease?**

| Disease | Diagnosed disease? | | Age of first diagnosis | Still on Treatment? | |
| --- | --- | --- | --- | --- | --- |
|  | Yes | No |  | Yes | No |
| Diabetes | 🞎 | 🞎 | 🞎 | 🞎 | 🞎 |
| Hypertension | 🞎 | 🞎 | 🞎 | 🞎 | 🞎 |

Section 5: Sleeping, mood & mental situation

**E1 During the past month, did you have any of the following**

**sleeping problem for ≥3days each week?**

| sleeping problem | Yes | No |
| --- | --- | --- |
| Taking >30 minutes to fall asleep after going to bed or waking up in the middle of the night |  |  |
| Waking up early and not being able to go back to sleep |  |  |
| The quality of sleep has adversely affect your daytime performance or activities |  |  |

**E2 Social support and social capital**

|  | strongly disagree | Disagree | Neutral | Agree | strongly agree |
| --- | --- | --- | --- | --- | --- |
| You receive emotional support from family | 🞎 | 🞎 | 🞎 | 🞎 | 🞎 |
| You always receive financial support from family | 🞎 | 🞎 | 🞎 | 🞎 | 🞎 |
| You frequently participated in activities organized by community organizations in the last year | 🞎 | 🞎 | 🞎 | 🞎 | 🞎 |
| You always received support from community organizations in the last year | 🞎 | 🞎 | 🞎 | 🞎 | 🞎 |
| You have been treated fairly by society | 🞎 | 🞎 | 🞎 | 🞎 | 🞎 |
