# Supplementary material for: Social capital and sleep disorders in Tibet, China
Source: BMC Public Health. 2021 Mar 25;21:591. doi: 10.1186/s12889-021-10626-x (PMC7992333; doi:10.1186/s12889-021-10626-x)
Supplement: Supplementary file 2 — Additional file 2: Supplementary File 2. Measurements of the social capital of the participants. [file 12889_2021_10626_MOESM2_ESM.docx]

**Supplementary File 2.** Measurements of the social capital of the participants

| Variables | Median (Q25, Q75) |
| --- | --- |
| **Family social capital** |  |
| You receive emotional support from family | 4 (4, 5) |
| You always receive financial support from family | 4 (4, 5) |
| **Total score** | 8 (7, 10) |
| **Community and society social capital** |  |
| You frequently participated in activities organized by community organizations in the last year | 2 (2, 2) |
| You always received support from community organizations in the last year | 4 (3, 5) |
| You have been treated fairly by society | 4 (4, 5) |
| **Total score** | 10 (9, 12) |
