# Supplementary material for: Social capital and sleep disorders in Tibet, China
Source: BMC Public Health. 2021 Mar 25;21:591. doi: 10.1186/s12889-021-10626-x (PMC7992333; doi:10.1186/s12889-021-10626-x)
Supplement: Supplementary file 3 — Additional file 3: Supplementary File 3. Conditions of sleep disorders of the participants. [file 12889_2021_10626_MOESM3_ESM.docx]

**Supplementary File 3.** Conditions of sleep disorders of the participants

| Variable | N | Percentage (%) |
| --- | --- | --- |
| Disorders of initiating and maintaining sleep |  |  |
| Yes | 969 | 30.0 |
| No | 2,219 | 69.6 |
| Early morning awakening |  |  |
| Yes | 862 | 27.0 |
| No | 2,326 | 73.0 |
| Daytime functioning |  |  |
| Yes | 383 | 12.0 |
| No | 2,805 | 88.0 |
| **Sleep disorders** | 1,271 | 39.8 |
